# Iranian Pharmacists’ Knowledge, Attitude and Practice Regarding Counterfeit Drugs

**Published:** 2012

**Authors:** Shieda Shahverdi, Mirhamed Hajimiri, Farshad Pourmalek, Hassan Torkamandi, Kheirollah Gholami, Somayeh Hanafi, Nikinaz Ashrafi Shahmirzadi, Mohammadreza Javadi

**Affiliations:** a *School of Pharmacy, Tehran University of Medical Sciences, Tehran, Iran.*; b *School of Epidemiology, Tehran University of Medical Sciences, Tehran, Iran.*; c *Dr.Shariati Hospital, Tehran University of Medical Sciences, Tehran, Iran.*; d *Clinical Pharmacy Department, Pharmacy School, Tehran University of Medical Sciences, Tehran, Iran. *

**Keywords:** Counterfeit drugs, Iran, Knowledge, Attitude, Practice, Pharmacists

## Abstract

*Background *Awareness of pharmacists about counterfeit drugs is necessary for health improvement in community. The purpose of the present study is to assess the knowledge and measure the professional attitude and practice of Iranian pharmacist about counterfeit drugs. In August 2008, a knowledge, attitude and practice (KAP) study was performed in a national sample of 794 pharmacists who participated in an Iranian Pharmacist Association congress. A questionnaire was prepared to collect Demographic and professional characteristics, Knowledge, attitude and practice of pharmacists regarding counterfeit drugs. The mean percent of participants who answer each practice questions correctly is 13.62% and none of questions have more than 14.7% of correct answer, while the participants’ attitude towards the subject is at high level. None of demographic factors represented a significant relationship with knowledge and the only related parameters with attitude, were age and gender. Increasing age of pharmacists resulted in attitude improvement (p = 0.013) and women›s attitudes were better than men (p = 0.05).The only related parameters with practice, were the number of working hours per a week and attitude. Increasing the number of working hours per a week, resulted in decreasing the desirable practice (p = 0.041) and attitude also had a direct relationship with practice (p = 0.011). *Conclusion *The most important finding in the present study was the pharmacists› low knowledge and practice level about counterfeit drugs, while their attitude towards this subject was at a high level. The results point out the need for designing and implementing educational programs.

## Introduction

A practical and scientific definition for “counterfeit drug” has been the goal of numerous organizations in the world. World Health Organization (WHO) definition for counterfeit pharmaceutical is: “Pharmaceutical that intentionally carries a wrong label of identity and origin”. In other words, counterfeit pharmaceuticals are produced with lower quality, safety and efficacy than standards; this could be true about both brands and generics (1). Counterfeit drug could cause damage by a lack of active or the presence of a harmful ingredient (2).

Drug counterfeiting is a global problem; it has been reported in developing countries such as India, Peru, Niger, Nigeria, Southeast Asia and Pakistan as well as first world countries such as United States which resulted in establishing Counterfeit Drug Task Force (3-10). The problem of counterfeit drugs was addressed internationally in Nairobi at the WHO Conference of Experts on the Rational Use of Drugs in 1985 for the first time. The outcome of the conference was to establish an organization responsibile for achieving information and distributing data to governments describing the nature and magnitude of counterfeit drugs. WHO has highlighted the international profile of the problem but actions taken thus far to combat this problem have been insufficient (11-13).

Most of counterfeit reports on pharmaceuticals in industrial countries are related to novel and expensive medicines such as hormones, steroids and antihistamines which influence life style. In the other hand in developing countries most of counterfeit reports are about medicines used in life threatening diseases like Malaria, Tuberculosis and AIDS (14).

The first anti counterfeit network system called Rapid Alarm System (RAS) established in 2005 by ‘‘WHO’’. The member countries are committed to follow and instantly report illegal medicines distribution to RAS so that the other members can prevent their distribution (15).

In last few years there have been several unofficial and official reports on detecting counterfeit drugs in Iranian Pharmaceutical market. Geographically speaking, Iran is located in a transitional pathway to Europe from south Asian countries such as south Asia region which are important suppliers of counterfeit drugs. To prevent spread of counterfeit drugs in the country awareness of health care providers especially pharmacists is necessary for health care improvement in community; for that reason we started counterfeit drug Knowledge Attitude Practice study.

## Experimental

In August 2008 a Knowledge Attitude Practice (KAP) study performed in an Iranian Pharmacist Association congress. A questionnaire was designed to collect a) demographic data, b) professional characteristics; c) knowledge, attitude and practice of pharmacists regarding counterfeit drugs. Knowledge and practice questions were designed as multiply choice and written questions. Attitude questions were prepared based on likert scale. The questionnaire was pre-tested on a sample of 20 pharmacists to evaluate the reliability and clarity of the questions using Cronbach›s alpha coefficient. Test-retest reliability was examined on the same sample, after a week, via applying intra-cluster correlation coefficient calculation. The final questionnaire was used to collect data from the main population.

*Statistical analysis*

In analyzing the results of the main population sample for the knowledge and practice questions, scores of 1 and zero were assigned to true and false answers respectively. For the attitude questions the score from 1 to 5 were assigned, 1 represented the lowest and 5 represented the highest attitude. The numerical variables (*e.g*. the number of working hours per a week), were described numerically.

To calculate the summary variables of knowledge, attitude and practice individually, the internal consistency reliability of the questions was first calculated using cronbach’s alpha coefficient. If ignoring one or more questions caused an increase in cronbach’s alpha, then that (those) question (s) were put aside from further illative analysis and cronbach’s alpha was recalculated. Subsequently, the scores of answers to the questions in each series (knowledge, attitude and practice) were added together and the obtained variable was divided by its maximum score, so that it became a proportion of one.

Consequently, the three summary variables of knowledge, attitude and practice were achieved, which could possess an amount with in the range of zero to 1, for each person. To determine the effective factors on knowledge (the summary variable of knowledge), the independent variables with entry into the regression model, were used. Accordingly, in order to determine the effective factors on attitude, the summary variable of knowledge was added to the series of independent variables, and to determine the effective factors on practice, both knowledge and attitude variables were added to the series of independent variables. The statistical significance level was considered as 0.05.

## Results and Discussion

Of 794 distributed questionnaires among the participants, 734 were completed and the response ratio is 92.4%.

*Demographics information*

The age of the male population entering the study was 37 years. The majority of the population entered the study were male (57.3%). The median number of years passed their graduation, the number of professional activity hours per week and the years of professional experience were 11, 35 and 10 respectively. The participants› demographic and practice characteristics are listed in Table 1. The educational degree status of 726 repliers were as follows, 692 pharmacists (94.3 %) with Pharm.D.,19 pharmacists (2.5%) with PhD, 10 pharmacists had a bachelorette and 6 pharmacists had a master degree in pharmacy. The workplace of 734 repliers is listed in Table 1.

**Table 1:** Selected characteristics of the study Population

**Characteristics**	**Result**
Age(year) • mean (min-max)	37 (22-82)
Years passed since graduation (year) • mean (min-max)	11 (1-52)
Number of professional activity hours per week(hour) • mean (min-max)	35 (0-90)
Number of seniority years(year) • mean (min-max)	10 (1-50)
Work positions (n) • Community pharmacy • Hospital pharmacy • Ministry of health • Pharmaceutical company • Faculty members of universities • Others	492 (67%) 80 (10.9%) 34 (4.6%) 38 (5.2%) 4 (0.5%) 86 (11.7%)

*Knowledge, attitude and practice variables*

The data concerning the knowledge about counterfeit drug questions from 734 repliers are summarized in Table 2 in a descending order of true answers. According to this table none of questions related to “The selective method for the counterfeit drugs” have more than 21.5% correct answer. The question about “examples of drugs which are likely to be counterfeit” has the lowest correct answer.

**Table 2:** Percentage of quality of knowledge answers

**Knowledge variable**	**True answer**	**False answer**	**No answer**
	(%)	(%)	(%)
The selective method for the counterfeit drugs	21.5	61.6	16.9
Penalty for supplying products without manufacture or entrance license	19.1	59.3	21.7
The effective factors on producing counterfeit drugs	18.5	65.9	15.5
Examples of drugs which are likely to be counterfeit	11.6	39.9	48.5
Mean	17.7	56.7	25.6

The distribution of 734 repliers› answers to the attitude questions, in a descending order of the sum of answers representing positive and completely positive attitudes are shown in Table 3. Questions 1, 3 and 4 are about supply counterfeits drug in the exceptional circumstances. High percent of participants approved selling counterfeit drugs which are the same in packaging with the brand ones, have no significant therapeutic effects and during shortage periods, they are 69.4%, 72.8% and 53.5% respectively.

**Table 3:** Percentage of quality of attitude answers

**Attitude variable**	**Completely positive attitude (%)**	**positive attitude (%)**	**Halfway attitude (%)**	**Negative attitude (%)**	**Completely negative attitude (%)**	**No answerv (%)**
**Question 4**	42.78	29.97	11.04	5.31	2.32	8.58
**Question 1**	46.73	22.62	8.99	8.86	3.95	8.86
**Question 3**	29.43	24.11	18.94	14.31	5.18	8.04
**Question 2**	17.71	24.93	20.98	16.35	11.58	8.45
**Question 7**	12.40	24.52	19.75	19.62	13.35	10.35
**Question 5**	6.95	11.31	16.21	34.20	23.57	7.77
**Question 6**	1.50	2.59	10.63	36.78	41.01	7.49
**Mean**	22.50	20.01	15.22	19.35	14.42	8.51

Question 1: In exceptional cases it is fine to use counterfeit drugs which packed different from original ones.

Question 2: In the case of any adverse reaction caused by a counterfeit drug, the dispensing pharmacist is the major responsible.

Question 3: In the case of medication shortage, it’s fine to provide it from unofficial (not registered) supplying source.

Question 4: It is fine to dispense some counterfeit drugs which are not vital to treat diseases.

Question 5: Individual pharmacists’ intervention can prevent dispensing of counterfeit drugs.

Question 6: Educational programs can provide pharmacists enough knowledge to prevent dispensing of counterfeit drugs.

Question 7: More than 50% of pharmacies nationwide are dispensing counterfeit drugs.

Questions 2, 5 and 7 discuss pharmacists’ responsibility in prevention of adverse effect of counterfeit drugs, combat and supply of these drugs, respectively. Among participants, about 28% do not blame pharmacist in adverse drug events due to use of counterfeit drugs. Also 36.9% of participants believe that more than 50% of community pharmacies supply these kinds of medications. Surprisingly assessing the answers given for question 6, only 4% of participants believe that training courses may have affect on professional manner about this problem. The distribution of 734 repliers’ answers to the practice questions listed in Table 4.

**Table 4:** Percentage of quality of Practice answers

**Practice variables**	**True practice**	**False practice**	**No answer**
	**(%)**	**(%)**	**(%)**
A patient refers to your pharmacy, complaining about acute and serious side effects due to the regular consumption of a drug and says that these problems did not occur using the same drug with the old packing. Do you contribute these problems to the possibility of using a counterfeit drug?	14.17	17.98	67.85
Do pharmacists accept to distribute a drug if it is entered the country illegally?	14.44	28.75	56.81
Have your counterpart pharmacists, has ever encountered foreign drugs which had been proven to be counterfeit?	14.71	32.43	52.86
Do your counterpart pharmacists usually have economic exchange with sellers and suppliers of drugs without a manufacture license or distribution permit from the health ministry?	13.76	35.29	50.95
Do your counterpart pharmacists inform the authorities, if the suppliers of counterfeit drugs referred to them?	14.44	54.5	31.06
Have you ever passed any special training course about identifying counterfeit drugs and dealing with this problem?	10.08	79.7	10.22
Mean	13.62	41.42	44.96

*The effective factors on knowledge, attitude and practice*

For both knowledge and attitude, none of the independent variables represented a significant relationship with knowledge and the only related parameters with attitude, were age and gender. Increasing age resulted in attitude improvement (p = 0.013) and gender also had a marginal relationship (at 0.05 level) with attitude, so that women›s attitudes were better than that of men (p < 0.05). The only related parameters with practice were the number of working hours per week and attitude. Increasing the number of working hours per week, resulted in decreasing the desirable practice (p = 0.041) and attitude also had a direct relationship with practice (p = 0.011).

The most important finding in the present study was the pharmacists’ low knowledge and practice level about counterfeit drugs, while their attitude towards this subject was at a high level. In addition, there was indirect relationship between working hours and the pharmacists› knowledge and attitude toward the counterfeit drugs. On the other hand, the critical factor which had increasing effect on practice level was the level of appropriate attitude. Interpretation of current study’s findings must be done with respect to the biases and possible limitations of the study. The internal consistency of investigating knowledge questions was low which would be due to low number of knowledge questions in the final questionnaire. Considering the absolute number of attitude questions and their little internal consistency, it seems that the knowledge level is estimated lower than the real, while the people›s attitude towards the subject is at a very high level. Although there is maximum knowledge about the selective method for identifying counterfeit drugs, knowledge about the objective examples of drugs likely to be counterfeited, is at the lowest level. The attitude level towards the unexceptionable supply of counterfeit drugs is the highest. On the other hand, the attitude level towards the effectiveness of pharmacist’s individual practice or the usefulness of training in field of counterfeit drugs is at the lowest level compared with the other attitude questions.

Although it is reported in the sample study that the appropriate practice has a direct relationship with appropriate attitude but in real life situation the appropriate practice level is not the same as appropriate attitude level. In order to convert the appropriate attitude to the appropriate practice, supportive changes in people›s decision making structure and in the environment of making decision are required. These changes could include implementing appropriate rules and regulations in the field. Lower practice level of the pharmacists with higher working hours show that increasing the number of working hours lowers the standards and/or quality of practice. Investigating the origin of patients› problem which has developed after consuming a new pack of drug is the most appropriate Practice for discovering a counterfeit drug. Attending training courses about counterfeit drugs had the lowest rate in the practice among the pharmacies in the field. Exchanging counterfeit drug with suppliers and not informing the authorities about this practice was also reported among the pharmacists practicing in the field.

These findings suggest that there is a great need for training pharmacists in the field of counterfeit. Also there is a lack of appropriate legislation and vacuum of regulatory control against the supply of counterfeit drugs.

## Conclusions

In conclusion, the findings from this study extend the understanding of the knowledge, attitudes, and practice regarding counterfeit drugs among pharmacists and they point out the need for designing and implementing educational programs. This training should be teaching pharmacists how to identify these drugs and how to inform the responsible authorities about counterfeit drugs suppliers.
